# PROTOCOL: Residential energy efficiency interventions: An effectiveness systematic review

**DOI:** 10.1002/cl2.1205

**Published:** 2021-11-30

**Authors:** Miriam Berretta, Joshua Furgeson, Collins Zamawe, Ian Hamilton, Yue Wu, Paul J. Ferraro, Neal Haddaway, John Eyers

**Affiliations:** ^1^ Internationational Initiative for Impact Evaluation London UK; ^2^ UCL Energy Institute London UK; ^3^ Independent Consultant; ^4^ Johns Hopkins University Baltimore Maryland USA; ^5^ Stockholm Environment Institute Stockholm Sweden

## Abstract

This review aims to identify, appraise and synthesise the evidence available on the effectiveness of energy efficiency measure installations, including those bundled with behavioural interventions. The synthesis will estimate the overall impact of these interventions as well as examine possible causes of variation in impacts. We will also attempt to assess the cost‐effectiveness of residential energy efficiency interventions.

## 1 BACKGROUND

### 1.1 The problem, condition or issue

Scientists agree that human activities are causing widespread climate change, and that reducing carbon dioxide (CO_2_) emissions is crucial to mitigating the global environmental and health threats caused by climate change (Intergovernmental Panel on Climate Change [IPCC], 2014). For example, the IPCC recently found that limiting global warming to 1.5°C—the level necessary to reduce challenging impacts on ecosystems, human health, and well‐being—requires large emissions reductions and comprehensive social changes (IPCC, 2018).

Residential energy use creates substantial carbon emissions. The International Energy Agency (IEA) estimates that residential usage accounts for 22% of the overall global final energy use and 17% of emissions (IEA, 2019). In residential buildings, roughly 32% of energy consumption is used for space heating, 29% for cooking, 24% for water heating, and the remainder by appliances, lighting and cooling (Ürge‐Vorsatz et al., 2015).

Residential energy use, and the associated CO_2_ emissions, could be significantly reduced through residential energy efficiency interventions (REEIs) (Gowrishankar & Levin, 2017; Russell‐Bennett et al., 2019). For example, one study reported that more energy efficient buildings could eliminate 550 million metric tons of CO_2_ equivalent emissions annually by 2050 (Gowrishankar & Levin, 2017). In addition to reducing energy use and emissions, REEIs are widely recognised as improving health and well‐being, as well providing by microeconomic and macroeconomic benefits (Campbell et al., 2014; Shrubsole et al., 2014; Russell‐Bennett et al., 2019).

Despite the promise of REEIs, a recent review of four studies found that REEIs saved less energy than forecasted (J‐PAL, 2019). Currently, there is no conclusive evidence on how REEIs affect energy consumption and ultimately global emissions. Synthesising the available evidence on REEIs would provide useful information to inform energy strategy and policy design, implementation and financing decisions.

### 1.2 The intervention

Improved residential energy efficiency can be achieved through flexible strategies, such as insulation, heating and lighting upgrades, boiler replacements, and new windows (GABC/IEA/UNEP, 2020). REEIs can involve improvements in the building/dwelling envelope; upgrades in the technical building/dwelling systems, such as space heating and cooling (Filippidou et al., 2019); or mechanisms that facilitate the installations and their correct use. The European Investment Bank (EIB) invests in projects designed to install such REEIs.

In this review, residences include private or social houses such as blocks of flats (also known as apartment and/or condominium buildings), public housing, as well as single family detached or semi‐detached housing. REEIs refer to the installation of energy efficiency measures (EEMs) that alter the building/dwelling, as well as complementary interventions that aim to increase the uptake and persistence of EEMs (Russell‐Bennett et al., 2019; Willand et al., 2015). EEMs can involve improvements in the building/dwelling envelope or upgrades in the technical building/dwelling systems, such as space heating and cooling (Filippidou et al., 2019), or mechanisms that facilitate the installations and their correct use. Governments and other organisations often fully or partially subsidise interventions for low income households and sometimes the broader housing market (Jacobsen, 2019). In this synthesis, we focus on two types of REEIs: EEM installation with and without behavioural interventions.

#### 1.2.1 EEM installation

EEM installation includes the replacement and upgrades of heating and cooling systems, the installation of insulation, more efficient boilers and heating, ventilation, and air conditioning technologies, among others (EEM installation examples are included in Adan & Fuerst, 2016; Howden‐Chapman et al., 2007; Maher, 2013). EEM installation often involves “weatherization”, which increases energy efficiency by protecting the building from sunlight, wind and precipitation (examples of studies evaluating EEM installations are Fowlie et al., 2018; Pigg et al., 2018). EEM installations can be further categorised by the amount of household involvement:

- *Passive* measures, such as insulation, do not require households to adopt a particular behaviour once completed
- *Semipassive* measures, for instance upgraded windows and doors, require residents to follow some simple behaviours (for instance, closing windows and doors to keep the rooms warm/cool)
- *Active* measures require continued correct behaviour for effectiveness, for instance heating controls.

EEMs are often installed after energy audits, which provide households with information and recommendations on building upgrades, as well as applicable utility and state incentives (Taylor et al., 2014).

#### 1.2.2 EEM installation combined with behavioural interventions

These bundled interventions combine EEM installation with interventions that provide information designed to change household behaviour. Behavioural interventions inform households on how to best use installed EEMs, such as advising households on how to set thermostats or how to reduce air conditioning load (examples of studies evaluating EEM installation in combination with behavioural interventions are Fowlie et al., 2018; James & Ambrose, 2017; Zivin & Novan, 2016). This guidance can be provided, for instance, by energy audits or other forms of technical assistance. Such guidance can be especially impactful for semiactive and active EEMs.

### 1.3 How the intervention might work

**Figure 1:**
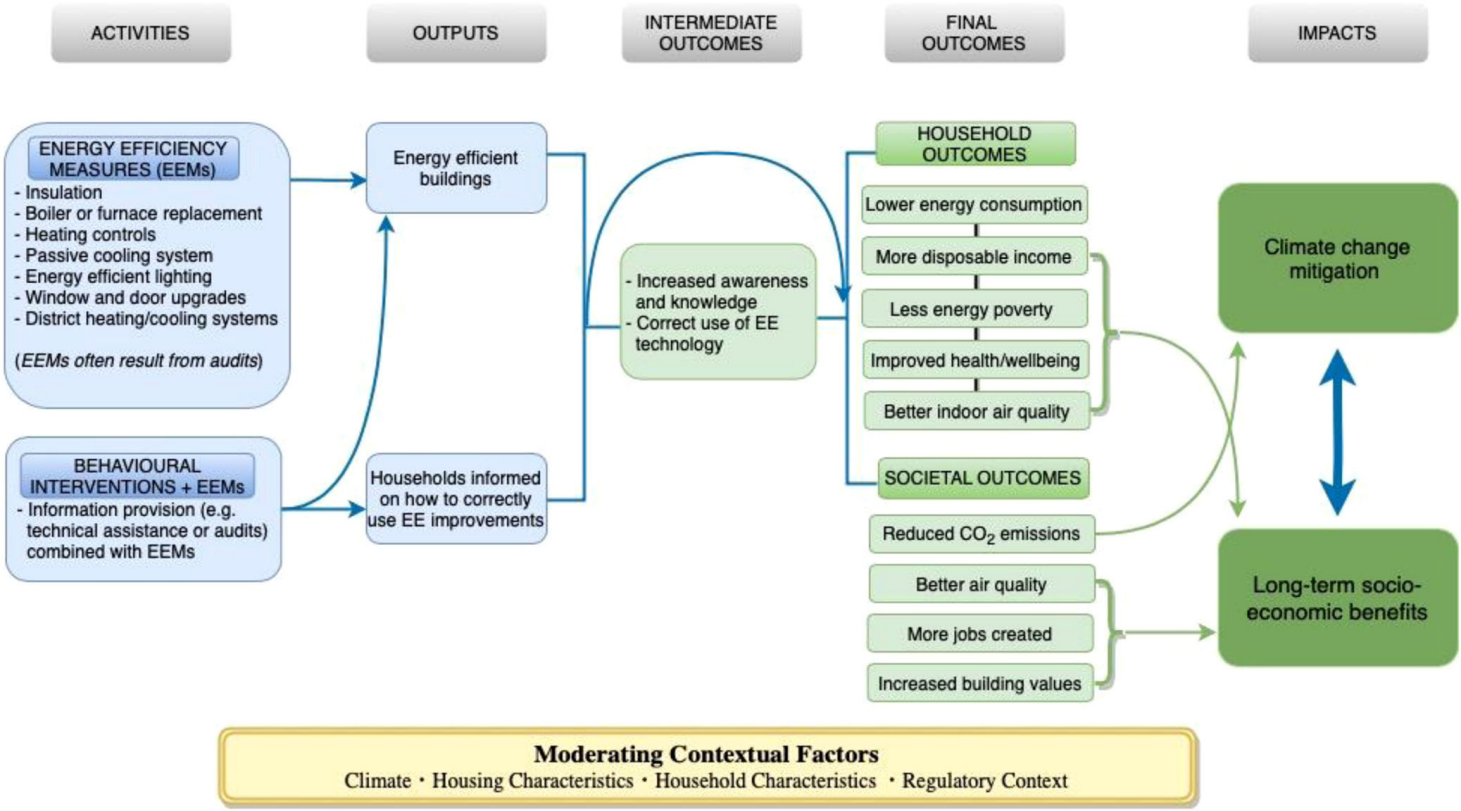
Theory of change. *Source*: 3ie, authors

After consulting relevant literature and experts, the review team developed a theory of change that shows how REEIs in single‐ and multifamily buildings can lead to climate change mitigation and long‐term socioeconomic benefits (Figure 1).

Starting from the left side of Figure 1, the activities column includes the interventions which will be studied in this review: the installation of EEMs with and without behavioural interventions. EEM installation programs typically include multiple EEMs and usually include some type of insulation and replacements of boilers, windows, and doors (Adan & Fuerst, 2016; Howden‐Chapman et al., 2007; Pigg et al., 2018). These installations often result from energy audits which identify relevant and cost‐effective upgrades (i.e., the audit directly leads to EEMs). Audits can also provide guidance on how to use installed EEMs, and so can also be a behaviour intervention.

Assuming the installation has been done correctly, the output should be a more energy‐efficient dwelling. If the intervention includes some form of information provision, a household should also understand how the implemented EEMs work and how to use them.

The intermediate outcomes include increased knowledge and awareness on how to reduce energy consumption, and behavioural changes such as correctly using and maintaining the technologies. Note that the intermediate outcomes do not necessarily lead to the final outcomes. In some cases, EEMs like insulation, are completely passive, and so the outputs lead directly to the final outcomes.

In this theory of change, we have categorised final outcomes as occurring at either the household level or societal level. At the household level, interventions can reduce energy consumption, thereby increasing disposable income, which leads to less energy poverty (lack of access to sufficient energy), ultimately resulting in improved household health and well‐being. In addition, interventions might also lead to better indoor air quality due to, for instance, better ventilation systems (Campbell et al., 2014; Grey et al., 2017; James & Ambrose, 2017; Russell‐Bennett et al., 2019; Shrestha et al., 2019). This sequential process is displayed by vertical black lines between the listed outcomes in Figure 1. At the societal level, there are reductions in global CO_2_ emissions, improved outdoor air quality, an increase in the number of jobs created due to the installation of EEMs, and an increase in value of the building stock (Campbell et al., 2014; Filippidou et al., 2019; Russell‐Bennett et al., 2019).

Ultimately, these outcomes are expected to lead to two long‐term impacts on society. First, the reduction of greenhouse gas emissions will mitigate climate change. Second, the outcomes will have long‐term socioeconomic impacts, such as: increased well‐being, especially for low‐income households who can use energy services continuously; reduced sickness and mortality rate due to less pollution and warmer homes with a subsequent reduced burden on the health sector; and direct and indirect effects on the economy through, for instance, increased GDP and increased tax revenues (Campbell et al., 2014).

The effects of REEIs can vary depending on the context (Russell‐Bennett et al., 2019); therefore, we include moderator factors to account for those differences. These include the characteristics of the housing (such as age), the average temperature of the location, the policies and standards of each context, and the characteristics and poverty status of the households.

Figure 1 presents the desired theory of change, but REEIs are complex interventions involving many different actors (such as installers and beneficiaries), and consequently some REEIs might lead to negative outcomes (Bone et al., 2010; Shrubsole et al., 2014). For instance, simply adding insulation without adjusting ventilation can reduce air circulation and the additional moisture can lead to mould and increases in other indoor‐generated pollutants (Shrubsole et al., 2014). Similarly, increased awareness and proper usage (intermediate outcomes) might cause increased energy usage if households feel that their good behaviour allows increased energy consumption in other areas (moral licensing, see Jacobsen et al., 2012; Tiefenbeck et al., 2013). Finally, REEIs might increase energy consumption due to the “rebound effect” of affordability. This happens when EEMs: (a) reduce the cost of operating equipment, causing the equipment to be used more, or (b) EEMs save households money and households use the additional income to increase energy consumption (Davis et al., 2014; Shrubsole et al., 2014). Therefore, simply considering energy consumption might underestimate utility gains from implementing these interventions (Allcott & Greenstone, 2012).

### 1.4 Why it is important to do this review

Large investments are being made in building energy efficiency. In 2019, roughly US$150 billion was invested in energy efficiency in the building sector globally (IEA, 2020). The EIB spent €4.6 billion on energy efficiency projects in Europe and around the world in 2019 (EIB, 2020). Energy efficiency building upgrades are also a sector of interest to major climate change funders like the World Bank and EIB.

3ie recently conducted an evidence gap map (EGM) on energy efficiency interventions which identified a cluster of impact evaluations examining REEI interventions (Berretta et al., forthcoming). Several impact evaluations found that REEIs can reduce demand for electricity, natural gas and heating oil, and ultimately contribute to reduced emissions and improved health (see for instance Koirala et al., 2013; Maidment et al., 2014). However, the estimated effects varied across studies. The proposed systematic review (SR) will synthesise this literature to estimate an average effect, and examine how that effect differs across subgroups. This information can inform energy efficient policies, strategies and investments globally.

The EGM identified three SRs that covered REEIs (Maidment et al., 2014; Munton et al., 2014; Willand et al., 2015), but each has limitations. Munton et al. and Willand et al. do not synthesise the effects reported in the included studies, but rather describe the evidence base and identify possible characteristics of effective interventions. The Maidment et al. review focuses on health outcomes and hence is limited in scope. Moreover, because of their methodological limitations, the quality appraisal in the EGM did not have “high confidence” in the findings of any of these SRs.

A few other recent SRs examining household energy efficiency interventions were not included in the EGM because they were not available at the time of the search; these SRs also had some limitations. Kerr and Winskel (2020) explored how public policy can encourage investment in energy efficient retrofits, but did not assess the effects of the interventions. Another recent review (Russell‐Bennett et al., 2019) explored how intervention characteristics (such as target population and design) influence effectiveness in Australia. The review had important limitations: the literature search was not comprehensive and the authors did not describe their approach to risk of bias and data synthesis.

Our SR has been commissioned by the Independent Evaluation Division of the EIB group, and the focus aligns with the EIB's climate action and environmental sustainability priorities. Specifically, REEIs are one of the EIB's priority areas as described in the EIB Energy Lending Policy and closely linked to the European Commission's Renovation Wave Strategy announced in October 2020 (European Commission, 2020).

## 2 OBJECTIVES

This review aims to identify, appraise and synthesise the evidence available on the effectiveness of EEM installations, including those bundled with behavioural interventions. The synthesis will estimate the overall impact of these interventions as well as examine possible causes of variation in impacts. We will also attempt to assess the cost‐effectiveness of REEIs.

We aim to answer the following research questions:

1. What are the effects of interventions that aim to reduce energy consumption in residential buildings?
2. To what extent do these effects vary by population group and location?
3. What factors relating to programme design, implementation, context and funding mechanisms are associated with better or worse outcomes?
4. What evidence is available on programme costs and incremental cost effectiveness in the included studies?

## 3 METHODS

### 3.1 Criteria for considering studies for this review

#### 3.1.1 Types of studies

To answer the first three research questions, we will include counterfactual studies that use an experimental or quasi‐experimental design and/or analysis method. We will include randomised and nonrandomised studies that aim to control for confounding and selection bias.

Specifically, we will include the following study types:

1. Randomised controlled trials (RCTs) with assignment at the individual, household, community or other cluster level, and quasi‐RCTs using prospective methods of assignment such as alternation.
2. Nonrandomised designs with either a known assignment variable(s) or a seemingly random assignment process:
  a. Regression discontinuity designs, where assignment is based on a threshold measured before intervention, and the study uses prospective or retrospective approaches of analysis to control for unobservable confounding.
  b. Natural experiments with clearly defined intervention and comparison groups which exploit apparently random natural variation in assignment (such as a lottery) or random errors in implementation, and so forth.
3. Nonrandomised studies with preintervention and postintervention outcome data for both intervention and comparison groups, where data are individual level panel or pseudo‐panels (repeated cross‐sections), which use the following methods to control for confounding:
  a. Studies controlling for time‐invariant unobservable confounding, including difference‐in‐differences, fixed‐effects models, or models with an interaction term between time and intervention for preintervention and postintervention observations.
  b. Studies assessing changes in trends in outcomes over a series of time points with a contemporaneous comparison (controlled interrupted time series), and with sufficient observations to establish a trend and control for effects on outcomes due to factors other than the intervention (such as seasonality).
4. Nonrandomised studies with a similar comparison group that control for observable confounding, including statistical matching, covariate matching, coarsened‐exact matching, propensity score matching, and multiple regression analysis.
5. Nonrandomised studies that control for confounding using instrumental variable approaches such as two‐stage least squares procedures.

#### 3.1.2 Types of participants

We will include any study that focused on households living in single‐ or multifamily residential buildings (dwellings) regardless of income or geographic location.

We will exclude any study focused on public, commercial, office or industrial buildings (the energy efficiency EGM only identified three studies targeting public commercial, office or industrial buildings). If a study includes residential and nonresidential buildings and reports separate estimates for residential buildings, the residential estimates are eligible.

#### 3.1.3 Types of interventions

We will include studies that measure the impact of at least one of the interventions listed in Table 1.

**Table 1:** Eligible interventions

Category	Intervention	Sample studies that examine the intervention
EEMs (*interventions can be combined*)	Wall/roof/floor cavity insulation	Adan and Fuerst (2016); Coyne et al. (2018); Curl and Kearns (2017); Hamilton et al. (2013); Howden‐Chapman et al. (2011); Scheer et al. (2013)
	Loft/attic insulation	Adan and Fuerst (2016); Coyne et al. (2018); Hamilton et al. (2013)
	External/internal wall insulation	Coyne et al. (2018); Davis et al. (2018); Grey et al. (2017); Maher (2013)
	Replacement (oil or gas) boiler or furnace	Adan and Fuerst (2016); Hamilton et al. (2013); Scheer et al. (2013)
	Heating controls	Coyne et al. (2018); Grey et al. (2017); Maher (2013)
	Passive cooling system and design	Davis et al. (2018)
	EE lighting (i.e. CFL, LED)	Coyne et al. (2018)
	Window and door upgrades	Coyne et al. (2018); Davis et al. (2018); Hamilton et al. (2013); Howden‐Chapman et al. (2011); Maher (2013)
	District heating/cooling systems	None identified
Behavioural interventions + EEMs	Information provision (e.g., audits) + EE improvements	James and Ambrose (2017); Fowlie et al. (2018); Zivin and Novan (2016)

*Note*: The studies that examine the intervention are those identified by the EE EGM.

#### 3.1.4 Types of outcome measures

##### Primary outcomes

We will include studies that measure at least one of the primary outcomes listed in Table 2. The primary outcomes measure energy consumption, and energy affordability, CO_2_ emissions and air quality indices and pollution rates. Because we are most interested in the effect of EEM on outcomes linked to climate change, at least one of the primary outcomes must be reported in a study for it to be included.

**Table 2:** Eligible outcomes

Level	Outcome category	Description
Primary outcomes	Net energy savings or consumption changes	Net energy (including fuel) or demand savings refer to the portion of gross savings that is attributable to the programme. This measurement involves separating out impacts that are a result of other influences, such as consumer self‐motivation. Given the range of influences on consumers' energy consumption, attributing changes to one cause (i.e. a certain programme) can be complex
	Energy security	Energy (including fuel) security is defined as the uninterrupted availability of energy sources at an affordable price. In this context, an EE intervention might have increased energy security by reducing energy costs due to more efficiency technologies, for example
	GHG emissions	Carbon related emissions (CO_2_) and noncarbon related emissions such as methane (CH_4_), nitrous oxide (N_2_O) and fluorinated gases
	Air quality indices and pollution levels	Air pollution or greenhouse gases that would have been emitted if more energy had been consumed in the absence of the EE programme. These emissions can be from the combustion of fuels at an electrical power plant or from combustion of heating fuels, such as natural gas or fuel oil at a project site
Secondary outcomes	Income savings	Increased economic savings due to more efficient new or upgraded equipment, or changed energy saving behaviour (e.g., bill savings)
	Health status, comfort, and wellbeing	Better health and quality of life resulting from the adoption of EE technologies or practices that improve the living environment, such as reducing the air pollution rate, decreasing rate of illnesses
	Job creation	New job creation due to the installation of new equipment or adoption of innovative practices that require more expert personnel or simply additional workers
	Building stock value	Increased property value due to the installation of new equipment or renovation of equipment

##### Secondary outcomes

Because EE interventions have multiple benefits (Campbell et al., 2014), we will also look at secondary outcomes in health, well‐being, economics, and behavioural outcomes. With guidance from the external advisory group and the internal EIB reference group, we will consider including additional outcomes of interest identified during the analysis.

##### Duration of follow‐up

We will include any follow‐up duration, coding multiple outcomes where studies report multiple follow‐ups.

##### Types of settings

We will accept studies from any type of setting and any part of the world. We will only review studies conducted in real‐world settings (i.e., we will not include efficacy studies).

### 3.2 Search methods for identification of studies

To reduce the risk of publication bias and identify relevant available evidence, we will conduct a comprehensive search for eligible published and unpublished studies. REEIs have improved incrementally and constantly over time. To include interventions most similar to those being implemented now, the search will be limited to studies published on or after January 1, 2000. No language restrictions will be placed on the searches; however, resource constraints might prevent inclusion of studies published in languages other than English.

#### 3.2.1 Electronic searches

We will run the search strategy in different academic databases. We will search the following databases:

- CAB Abst
- Econlit
- Greenfile
- Repec
- Academic Search Complete
- WB e‐lib
- WoS (SCI & SSCI).

We will also search the following organisational databases, which might include evidence on interventions in the energy sector:

- Collaboration for Environmental Evidence
- E2e, group of economists focused on EE and IEs
- eceee and ACEEE Summer Study
- BECC
- Energy consumers Australia
- Environmental and Energy Study Institute EESI
- eScholarship University of California
- GEF (Global Environmental Facility) evaluation database
- Institute for European Energy and climate policy
- Institute of the Environmental and sustainability
- International Energy Agency
- International Energy Program Evaluation Conference
- Energy Evaluation Conferences (which covers Europe and also Asia). https://energy-evaluation.org

Finally, we will search the following evaluation repositories:

- 3ie Repository of IEs
- 3ie RIDIE (Registry for International Development IEs)
- African Development Bank (AfDB)
- Asian Development Bank (ADB)
- BREAD
- CARE International
- Centre for Effective Global Action (CEGA)
- Centre for Public Impact
- DFID Research for Development Department (R4D)
- ICNL Research Centre
- IFPRI
- Independent Development Evaluation, AfDB
- Innovations for Poverty Action (IPA)
- Inter‐American Development Bank Publications
- IRC
- J‐Poverty Action Lab (J‐PAL)
- Locus (International Development Coalition)
- LSE Grantham Research Institute on Climate Change and the Environment
- Mercy Corps
- OECD iLibrary
- OpenGrey
- RTI International
- Samuel Hall (evaluations)
- The Campbell Collaboration Library
- Transparency International (TI):
- United Nations Evaluation Group
- USAID Development Clearing House
- World Vision.

#### 3.2.2 Searching other resources

We will also search for studies in the bibliography of the energy efficiency EGM and other relevant SRs and literature reviews. In addition, we will screen the reference lists of included studies and undertake forward citation‐tracking for those studies using Google Scholar.

To identify additional studies, we will contact key experts and organisations through our review external advisory group and internal EIB reference group.

##### Targeted search for studies addressing

To answer question 4 relating to programme design, implementation, financial mechanisms and context, we will attempt to identify programme and project documents associated with the programmes identified in the first stage of the search. We will do this by undertaking a targeted search for programme names and authors using Google. Evidence on context and mechanisms will be collected from all the included studies. Programme mechanisms may be suggested by study authors or identified by the review team.

### 3.3 Data collection and analysis

#### 3.3.1 Criteria for determination of independent findings

Estimation of a standard meta‐analytic effect size relies on the statistical assumption of independence of each included estimation of effect (Hedges, 2019). Dependent effect sizes arise when one study provides multiple results for the same outcome of interest, when a study has multiple treatment arms compared to the same comparison group, or multiple studies use the same data set and report on the same outcome. We will therefore use the following rules to ensure that only statistically independent effect sizes are included in any one meta‐analysis.

Where we identify several studies/publications that report on the same analysis we will use effect sizes from the most recent publication. If we identify more than one study using the same data set, or where multiple outcomes are reported using similar outcome constructs within the same study, to enhance the potential for meta‐analysis we will select the study or construct which is the most similar to other estimates for the same outcome type. However, we will extract data and calculate effect sizes for the other outcome constructs. Where different studies report on the same programme but use different samples (e.g., from different regions), we will include both estimates, treating them as independent samples, provided effect sizes are measured relative to separate control or comparison groups.

If one study reports multiple effect size estimates using different specifications for the same outcome, we will choose the one with the lowest assessed risk of bias.

If studies report more than one follow up period for one outcome, we will identify the most common follow‐up period and include the follow up measures that match this most closely in the meta‐analysis. However, we will extract data and calculate effect sizes for all time points and report these in the review.

If we identify studies with multiple treatment arms and only one comparison group, we will estimate an effect size for both arms, and either choose the effect estimate from the treatment arm that tests an intervention that most commonly resembles the other interventions included in the meta‐analysis to synthesise.

When studies use different assumptions to convert measured outcomes to projected outcomes (such as how reduced air pollution leads to improved health), we will use a single set of parameters to convert outcomes across all studies. We will also present the reported outcomes.

#### 3.3.2 Selection of studies

We will import all search results into EPPI‐Reviewer 4^1^ and remove duplicates. After testing the inclusion/exclusion criteria for operationalisability, all studies will be double screened against the review inclusion criteria using information available in the title and abstract by two independent research assistants, with any disagreements being resolved through conversations with a core review team member. Where a study's title and abstract do not include sufficient information to determine relevance, the study will be included for review at full text.

While undertaking title/abstract screening, we will take advantage of the text‐mining capabilities of EPPI‐Reviewer 4, to reduce the initial screening workload (O'Mara‐Eves et al., 2015). We will use the inclusion/exclusion classifier (O'Mara‐Eves et al., 2015; Thomas et al., 2011) in EPPI Reviewer 4. We will first screen around 5 percent of studies and reconcile them to “train” the classifier which will classify studies into groups based on their probability of inclusion in the review. To get more accurate results, we will repeat this process two or three times because the function continues to learn as screening progresses.

We will conduct piloting and verification of the machine learning functioning, and expect to be able to exclude studies with a low probability of inclusion (<20% probability of inclusion) automatically from the review. We will screen a random 10% sample of the automatically excluded studies as a check on accuracy of the function, in case we find even one abstract includable, then we will screen all of them.

Studies included for full‐text screening will then be double screened by two independent reviewers. Disagreements on inclusion or exclusion will be resolved by discussion with a core review team member and the input of an additional core reviewer if necessary. The screening of studies for inclusion under review question 4 will take place in a later stage of screening after studies have been identified for inclusion in the core effectiveness component of the review. The studies identified to answer question 4 will be assessed for relevance, that is, whether they cover one of the programmes included to answer research questions 1–3 and whether they provide information on the design, implementation processes, context or mechanisms at play.

#### 3.3.3 Data extraction and management

We will extract the following descriptive, methodological, and quantitative data from each included study using a standardised data extraction form (provisional form provided in Supporting Information Appendix 1):

- Descriptive data including authors, publication date and status as well as other information to characterise the study including country, type of intervention and outcome, population, context, type of intervention.
- Methodological information on study design, measurement and analysis methods, type of comparison (if relevant) and external validity (e.g., population and setting).
- Quantitative data for outcome measures, including outcome descriptive information, sample size in each intervention group, outcomes means and SDs, test statistics (e.g., *t* test, *F* test, *p* values, 95% confidence intervals), cost data, and so on.
- Information on intervention design, including how the interventions was funded and with which financial mechanisms, transparency and accountability characteristics, participant adherence, contextual factors and programme mechanisms.

We will extract quantitative data for synthesis using Excel. We will extract descriptive, methodological and qualitative data using Excel. Descriptive and qualitative data will be single coded by one reviewer and checked by a second reviewer.

#### 3.3.4 Assessment of risk of bias in included studies

We will assess the risk of bias for the eligible impact evaluations, drawing on the signalling questions in the 3ie risk of bias tool which covers both internal validity and statistical conclusion validity of experimental and quasi‐experimental designs (Waddington et al., 2012) and the bias domains and extensions to Cochrane's ROBINS‐I tool (Sterne et al., 2016). Two reviewers will independently assess the risk of bias. If there are disagreements, they will be resolved by discussion and the involvement of a third reviewer as necessary. The provisional risk of bias tool can be found in Supporting Information Appendix 2. We will conduct the risk of bias assessment at the study level, noting any potential differences in methods and risk of bias for different outcomes.

We will assess risk of bias based on the following criteria, coding each study as “Yes”, “Probably Yes”, “Probably No”, “No” and “No Information” according to how they address each domain:

- Factors relating to baseline confounding and biases arising from differential selection into and out of the study (attrition);
- Factors relating to biases due to deviations from intended interventions (e.g., performance bias and survey effects) and motivation bias (Hawthorne effects);
- Factors relating to biases in outcomes data collection (e.g., social desirability or courtesy bias, recall bias);
- Factors relating to biases in reporting of analysis.

We will report the results of the assessment for each of the assessed criteria for each study in a table.

In addition, we will explore if there are systematic differences in outcomes between primary studies with different risk of bias. If meta‐analysis is feasible, we will conduct sensitivity analysis to assess the robustness of the results to the risk of bias in included studies.

#### 3.3.5 Measures of treatment effect

Studies examining similar outcomes might report effects using different metrics. To enable a synthesis of these findings, where possible, all study effects will be converted to standardised effect sizes that express the magnitude or strength of the relationship between the intervention and outcome (Borenstein & Hedges, 2019; Cooper et al., 2019).

For continuous outcomes comparing group means in a treatment and control group, we will calculate the standardised mean difference (SMDs), or Hedges *g*, its variance and SE using formulae provided in Cooper et al. (2019). An SMD is the difference in means between the treatment and control groups divided by the pooled SD of the outcome measure. Cohen's *d* can be biased in cases where sample sizes are small. Therefore, we will always adjust Cohen's *d* to Hedges *g* using the following formula:

$$g≅d\left(1-\frac{3}{4(n_{1}+n_{2})-9}\right).$$

For studies reporting regression results, we will follow the approach suggested by Keef and Roberts (2004) using the regression coefficient and the pooled SD of the outcome.

Where outcomes are reported in proportions of individuals, we will calculate the Cox‐transformed log odds ratio effect size (Sánchez‐Meca et al., 2003):

$$d=\frac{\ln(\mathrm{OR})}{1.65},$$

where OR is the odds ratio calculated from the two‐by‐two frequency table.

#### 3.3.6 Unit of analysis issues

Unit of analysis errors can arise when the unit of allocation (assignment) of an intervention is different from the unit of analysis of the study, and this is not accounted for in the analysis. We will assess studies for unit of analysis errors, and, if unit of analysis errors exist, we will correct for this by adjusting the SEs (Hedges et al., 2010; Higgins et al., 2011):

(d)′=(d)*1+(m−1)c,

where *m* is the average number of observations per cluster and *c* is the intra‐cluster correlation coefficient. Where included studies use robust Huber‐White SEs to correct for clustering, we will calculate the SE of *d* by dividing *d* by the *t* statistic on the coefficient of interest.

#### 3.3.7 Dealing with missing data

In cases of relevant missing or incomplete data needed for meta‐analysis (such as means and SDs), we will contact study authors to obtain the required information. If we are unable to obtain the necessary data, we will report the characteristics of the study and state that it could not be included in the meta‐analysis or reporting of effect sizes due to missing data.

#### 3.3.8 Assessment of reporting biases

We will attempt to reduce publication bias by searching for and including grey literature in the review (e.g., forward citation‐tracking in Google Scholar). We will also undertake exploratory tests for the presence of publication bias with funnel plots (Egger et al., 1997).

#### 3.3.9 Data synthesis

Once we have identified all the included studies, we will map out designs, interventions, comparisons, and outcome measures. Based on an examination of these characteristics, we will determine how best to synthesise findings across studies.

We will only synthesise studies using meta‐analysis when we identify at least two effect sizes involving a similar intervention, outcome and comparison group. We provisionally plan to analyse studies in the same meta‐analysis when they evaluate the same intervention (e.g., loft/attic insulation), or the same combination of interventions (e.g., loft/attic insulation and heating controls). However, once all eligible interventions are identified, in consultation with the advisory group, we might decide to combine similar interventions. We might not combine studies examining the same intervention if there are important differences in implementation due to subsidy variation (i.e., high nonparticipation in the treatment condition or high contamination in the control group). Finally, separate analyses might be necessary when some studies have a “pre‐bound effect”, the overestimation of households' consumption rates before the EEMs are implemented, leading to underestimation of the EEMs actual impacts (Sunikka‐Blank & Galvin, 2012).

We expect settings, intervention characteristics, and other relevant factors to vary across studies, and plan to use a random‐effects meta‐analysis. We will use the *metafor* package in R (R Development Core Team, 2018) to conduct the meta‐analysis (Viechtbauer, 2010).

When there are insufficient studies with similar interventions, outcomes, and comparisons, we will describe and synthesise findings narratively, including tables reporting findings from all studies.

#### 3.3.10 Subgroup analysis and investigation of heterogeneity

If sufficient data are available from included studies, we will conduct subgroup analysis for the following categories of interest to the primary funder:

- Resident socioeconomic status
- Region of residency (European Union‐27 and the UK vs. other)
- The source of the funds used for the intervention, and if applicable, which kind of financial instrument
- Climatic region (if there are sufficient resources and time).

We will assess heterogeneity by calculating the *Q* statistic, *I* ^2^, and *τ* ^2^ to provide an estimate of the amount of variability in the distribution of the true effect sizes (Cooper et al., 2019). We will complement this with a graphical presentation of heterogeneity of effect sizes using forest plots.

#### 3.3.11 Sensitivity analysis

We will conduct sensitivity analysis to assess whether the results of the meta‐analysis are sensitive to the removal of any single study (e.g., by using the leave1out command in R). We will also assess sensitivity of results to inclusion of high risk of bias studies by removing these studies from the meta‐analysis and comparing results to the main meta‐analysis results.

## ADVISORY GROUP MEMBERS

Dr. Gesche Huebner—University College London.

Dr. James Milner—London School of Hygiene and Tropical Medicine.

Dr. Nicola Willand—Royal Melbourne Institute of Technology and Melbourne Technical College.

Marina Economidou—European Commission.

Claire Walsh—J‐PAL, King Climate Action Initiative.

Jan Minx—Mercator Research Centre on Global Commons and Climate Change.

## CONTRIBUTIONS OF AUTHORS

*Content*: Ian Hamilton, Miriam Berretta, and Yue Wu.

*Systematic review methods*: Joshua Furgeson, Collins Zamawe, Miriam Berretta, and Neal Haddaway.

*Statistical analysis*: Joshua Furgeson.

*Information retrieval*: John Eyers.

## DECLARATIONS OF INTEREST

There are no potential conflicts of interest.

## SOURCES OF SUPPORT

**External Sources**

European Investment Bank.

## Supporting information

Supporting information.

## Footnotes

1 EPPI‐Reviewer 4 is a web‐based software program for managing and analysing data in literature reviews. It has been developed for all types of SR (meta‐analysis, framework synthesis, thematic synthesis, and so on). It manages references, stores PDF files and facilitates qualitative and quantitative analyses such as meta‐analysis and thematic synthesis. It also contains some new “text mining” technology which aims to make systematic reviewing more efficient.
