# Supplementary material for: PROTOCOL: Residential energy efficiency interventions: An effectiveness systematic review
Source: Campbell Syst Rev. 2021 Nov 30;17(4):e1205. doi: 10.1002/cl2.1205 (PMC8988823; doi:10.1002/cl2.1205)
Supplement: Supplementary file 1 — Supporting information. [file CL2-17-e1205-s001.docx]

# Appendices

**Appendix A: Data Extraction Template**

*Note: this coding tool may be adapted after further piloting.*

**Descriptive / qualitative coding tool**

| **Description** |  | **Question** | **Coding** |
| --- | --- | --- | --- |
| **Section A: Core key words** | | | |
| **Unique study ID** | - | What is the EPPI ID for the record? | For example, 44126990 |
| **Reviewer name** |  | Who completed the data extraction? | Open answer |
| **Document type** | - | Is this the main paper of a linked document? | Report 'main paper' or 'linked document' |
| **Main paper study ID** |  | If linked document, provide EPPI ID for the main paper | ID number |
| **Time taken to complete extraction** |  | How many minutes did it take to code the study?   We are genuinely interested in understanding how long it takes. We completely understand that some studies take longer to code. There are no repercussions for reporting any length of time so try to report truthfully - this helps us with planning future work, and means we budget correctly to involve consultant teams. | Mins |
| **Section B: Study characteristics** | | | |
| **Form of publication** |  | What is the impact evaluation publication type? | 1 = Peer-reviewed journal |
|  |  |  | 2 = Book chapter/book |
|  |  |  | 3 = Conference paper |
|  |  |  | 4 = Organisation report 5 = Working-paper |
|  |  |  | 6 = Implementation document |
|  |  |  | 7 = Website |
|  |  |  | 8 = Other grey (specify) |
|  |  |  | 9 = PhD thesis / dissertation |
| **Publication year** |  | What is the publication year? | XXXX |
| **Study funding / Funding agency** |  | Who is funding the evaluation/study? | 1 = Public institution (e.g. gov, university, research institute) |
|  |  |  | 2 = Private institution (e.g. private firm) |
|  |  |  | 3 = Multilateral Organisation (e.g. World Bank, UN) |
|  |  |  | 4 = Foundations |
|  |  |  | 5 = NGO |
|  |  |  | 999 = Not clear |
|  |  |  | 9 = Not applicable (Non-funded) |
| **Name of study funding agency** |  | Please add name of the agency funding the evaluation | Open answer |
| **Study setting (World Bank)** |  | Select the World Bank income classification of the country at the time of the study | 1 = Low income country |
|  |  |  | 2 = Lower-middle income country |
|  |  |  | 3 = Upper-middle income country |
|  |  |  | 4 = High income country |
| **Region (World Bank)** |  | Select region(s) the study was conducted in according to World Bank. For more info on region classification see http://data.worldbank.org/country | 1 = East Asia & Pacific |
|  |  |  | 2 = Europe & Central Asia |
|  |  |  | 3 = Latin America & Caribbean |
|  |  |  | 4 = Middle East & North Africa |
|  |  |  | 5 = South Asia |
|  |  |  | 6 = North America |
|  |  |  | 7 = Sub-Saharan Africa |
| **Country** |  | List country/countries the study was conducted in | Country 1, Country 2, etc. |
| **Detailed location** |  | If provided, give detailed information on where the study took place within a country, for example regions/districts covered | Open answer |
| **Independence of evaluation** |  | What level of independence is there between the implementing agency and study team? | 1 = Funding and author team independent of implementers/ funders of programme.  i.e. The organisation that funded the evaluation, and the evaluation authors, are independent from those responsible for implementing the programme. |
|  |  |  | 2 = Funding independent of implementers/ funders of programme, but includes authors from funder/ implementer  i.e. The evaluation was not funded by those responsible for implementing the programme. But the evaluation includes authors from either the funding agency of the implementation team. |
|  |  |  | 3 = Evaluation funded and undertaken by funders/ implementers.  i.e. The evaluation was funded and undertaken by those responsible for implementing the programme. |
|  |  |  | 8 = Unclear |
| **Independent data collection** | - | Has the data been collected by an independent organisation (a different one from the implementer organisation)? | 1 = Yes |
|  |  |  | 2 = No |
|  |  |  | 8 = Not clear |
| **Conflict of interest** | - | Is there a potential conflict of interest associated with study which could influence results collected/reported? (e.g. Is there a declaration of conflict of interest?  Is any of the authors related in any way to the funding or implementing institution?) | 1 = Yes |
|  |  |  | 2 = No |
| **Comments on conflict of interest** | - | Please add reason for your answer to whether there is a conflict of interest. | Open answer |
| **Language of publication** | - | Language of publication of the impact evaluation, e.g. Spanish, English etc. | Open answer |
| **Other methods** | - | If the impact evaluation addresses other questions than effectiveness - note questions and methods used here. | Open answer (this will include for example mixed-methods to assess implementation, adherence, participant views etc.) |
| **Baseline socio-economic status of participants** | - | Report any data / description on baseline socio-economic status of participants. | Open answer |
| **Section C: Study sample characteristics** | | | |
| **Unit of observation** |  | What is the unit of observation for the analysis? | Open answer, include whether group are residential, commercial or community. |
| **Total number of units** |  | State the total number of units, if possible by treatment and control groups. | Open answer |
| **Age groups within sample** |  | What is the age group of respondents within the sample?   When the unit of analysis is not an individual, report the most common group, e.g. a school would focus on children. | 1 = Children <18 years |
|  |  |  | 2 = Adults ≥18 |
|  |  |  | 3 = Both children and adults |
|  |  |  | 8 = Not clear |
|  |  |  | 9 = Not applicable |
| **Sex** |  | What is the sex of the sample? | 1 = Males only |
|  |  |  | 2 = Females only |
|  |  |  | 3 = Mixed |
|  |  |  | 8 = Not clear |
|  |  |  | 9 = Not applicable |
| **Income level** |  | Are the beneficiaries low income households? | 1= yes 2 = no 999 = Unclear |
| **Other useful information** |  | Report any other characteristics relating to the study sample. | Open answer |
| **Section D: Intervention characteristics** | | | |
| **Control/comparison scenario** |  | What intervention did the control/comparison group receive? | 1 = No treatment |
|  |  |  | 2 = Business as usual (specify) |
|  |  |  | 3 = Alternative intervention (specify) |
|  |  |  | 4 = Other (specify) |
|  |  |  | 999 = Not clear |
| **Intervention name** |  | State the programme or project name. If no name, then list the location | Open answer |
| **Intervention description** |  | Provide descriptive details about the intervention in your own words.  Aim to provide up to 2-3 sentences, although expect to provide more or less content depending on the complexity of the intervention. | Open answer |
| **Specific objectives of intervention** |  | State any objectives stated in study or other document.  Please directly copy and paste the objectives stated in the document. | Open answer |
| **Intervention activities** |  | Is the intervention comprised of one or more activities? | 1 = Single service / activity |
|  |  |  | 2 = Multiple services / activities |
|  |  |  | 999 = Unclear |
| **Intervention type** |  | What are the activities the intervention is comprised of? | 1 = Wall/roof/floor cavity insulation |
|  |  |  | 2 = Loft/attic insulation |
|  |  |  | 3 = External/internal wall insulation |
|  |  |  | 4 = Replacement (oil or gas) boiler or furnace |
|  |  |  | 5= Heating controls |
|  |  |  | 6 = Passive cooling system and design |
|  |  |  | 7 = EE lighting (i.e. CFL, LED) |
|  |  |  | 8 = Window and door upgrades |
|  |  |  | 9 = District heating/cooling systems |
|  |  |  | 10 = Energy audits + EE improvements |
|  |  |  | 11 = Behaviour change interventions+ EE improvements |
|  |  |  | 999 = Unclear |
|  |  |  |  |
| **Intervention funding agency** |  | Type of funder | 1=Government |
|  |  |  | 2=NGO |
|  |  |  | 3=Multilateral/bilateral organisation |
|  |  |  | 4= Foundation |
|  |  |  | 5= Private sector |
|  |  |  | 6= Other |
| **Intervention funding agency** |  | Name of intervention funding agency | Open answer |
| **Setting of the intervention** |  | Where is the intervention delivered? | 1 = Rural |
|  |  |  | 2 = Urban |
|  |  |  | 3 = Both |
|  |  |  | 999 = Unclear |
| **Intervention scale** |  | At what level is the intervention being delivered at? | 1 = National delivery |
|  |  |  | 2 = Regional (delivery in one or more regions) |
|  |  |  | 3 = Local level (delivery in one or more sub-regions) |
|  |  |  | 999 = Unclear |
| **Other useful information** |  | Report any other characteristics relating to the intervention. | Open answer |
| **Targeting methods** |  | How were beneficiaries selected for the programme (E.g.: how was the targeting implemented)? | Open answer |
| **Intervention start** |  | Start date of intervention, if no stated write 999 | XX/XXXX |
| **Intervention end** |  | State end date (if ongoing state ongoing) | XX/XXXX |
| **Intervention length** |  | Start intervention length (months) | State number of months |
| **Consideration of equity** |  | Does the study consider equity? | 1 = Yes 2 = No |
| **Equity methods** |  | Does the study explicitly consider equity in any of the following ways? | 1=  Intervention targets vulnerable population |
|  |  |  | 2 =  Sub-group analysis by sex |
|  |  |  | 3 =  Sub-group analysis (other than sex) |
|  |  |  | 4 =  Heterogeneity analysis (other than sub-group) |
|  |  |  | 5 =  Equity sensitive analytical framework |
|  |  |  | 6 =  Equity sensitive methodology |
|  |  |  | 7 =  Equity sensitive research process |
|  |  |  | 8 =  Measures effects on an inequality outcome |
|  |  |  | 9 = Research ethics informed by equity |
|  |  |  | 10 = Not applicable |
| **Equity dimension** | - | If answered 1-3 in "Equity methods" What dimension(s) of equity does the study consider? Report "N/A" otherwise.  PROGRESS + indicators (multiple choice - may pick more than one). For more information, please see:  https://www.jclinepi.com/article/S0895-4356(13)00334-X/pdf. | 1 = place of residence |
|  |  |  | 2 = race / ethnicity |
|  |  |  | 3 = occupation |
|  |  |  | 4 = sex |
|  |  |  | 5 = religion |
|  |  |  | 6 =education |
|  |  |  | 7 = socioeconomic status |
|  |  |  | 8 = social capital |
|  |  |  | 9 = age |
|  |  |  | 10 = disability |
|  |  |  | 11 = sexual orientation |
| **Information about program take-up** | - | Is there any information about program take-up?  Commentary by authors should be used when information on program take / up etc. is not backed up by some sort of research / when the authors do not report that/how they collected data to assess these areas. | Open answer. |
|  |  |  |  |
|  |  |  |  |
| **Methods of assessing take-up** | - | Which methods are used to assess program take-up? | 1= Observation by intervention staff |
|  |  |  | 2= Reporting by participants |
|  |  |  | 3= Other |
|  |  |  | 4= Commentary from author |
|  |  |  | 999= Not measured, N/A |
| **Results of the assessment of take-up** | - | What is the result/ information provided of the assessment of program take-up? If the programme implementation did not permit beneficiaries to accept the intervention then report this. | Open answer. |
| **Section E: Outcome domains** | | | |
| **Outcome type** |  | What outcomes were assessed? | 1 = Primary outcomes |
|  |  |  | 2 = Secondary outcomes |
| **Section F: Primary outcomes** | | | |
| **Net energy savings or consumption changes** |  | Were any of the following outcomes assessed (multi code okay)? | 1 = rates of energy saved |
|  |  |  | 2 = changes in consumption used |
|  |  |  | 3 = Other (specify) |
| **Energy security** |  | Were any of the following outcomes assessed (multi code okay)? | 4 = beneficiaries can now pay the energy bill |
|  |  |  | 5 = beneficiaries can afford to keep higher temperature in the houe/apartment in winter |
|  |  |  | 6 = Other (specify) |
| **GHG emissions** |  | Were any of the following outcomes assessed (multi code okay)? | 7= changes in GHG emissions rates |
| **Air quality indices and pollution (rates)** |  | Were any of the following outcomes assessed (multi code okay)? | 8 = changes in air quality rates in the house/apartment |
|  |  |  | 9 = changes in air quality rates in the area (e.g. city, region) |
|  |  |  | 10 = Other (specify) |
| **Section G: Secondary outcomes** | | | |
| **Income savings** |  | Were any of the following outcomes assessed (multi code okay)? | 1 = cheaper energy bills |
|  |  |  | 2 = Other (specify) |
| **Health status, comfort, and wellbeing** |  | Were any of the following outcomes assessed (multi code okay)? | 3 = Mortality rate |
|  |  |  | 4 = Sickness rate |
|  |  |  | 5 = Mental health rates |
|  |  |  | 6 = Other (specify) |
| **Job creation** |  | Were any of the following outcomes assessed (multi code okay)? | 7 = No. of jobs created |
|  |  |  | 8 = Other (specify) |
| **Building stock value** |  | Were any of the following outcomes assessed (multi code okay)? | 9 = Reported increases of the building/house value |
|  |  |  | 10 = Other (specify) |

**Quantitative data coding tool**

|  | Description | Question | Coding |
| --- | --- | --- | --- |
| ID | Unique study identification # |  | For example, SC001 |
|  | First author - impact evaluation | Surname | Open answer |
| Outcome for effect size (answer for all studies) | Outcome | Which outcome is being coded? | 1 = Knowledge and attitudes about services 2 = Access to services 3 = Use of services 4 = Performance or quality of services 5 = Income or poverty status 6 = Health outcome 7 = Nutritional status / food security 8 = Resilience (including coping strategies) 9 = Environmental (non-human) outcome 10 = Social and psychological outcomes 11 = Other |
|  | Definition of outcome | Please provide the authors definition of the outcome (including description of the sub-group if relevant) | Open answer |
|  | Follow up period | What is the follow up period of this outcome after initiation of intervention? (specify in months) | Number of months |
|  | Sub-group analysis | Is this effect size data for a sub-group? | 1 = No 2 = Yes |
|  | Sub-group analysis description | If yes to question 2, which type of sub-group? | Open answer - this can include separate samples for gender, income, place of residence |
|  | Effect size location | Which page(s) contain the effect size data? | Open answer |
|  | Data to be extracted | Which type of data to be extracted? | 1 = Continuous - means and SDs 2 = Continuous - mean difference and SD 2 = Dichotomous outcome - proportions 3 = Regression data |
| Effect size data (answer for all studies) | Sample size metric | Sample size unit of analysis | 1= Individual 2= Household  3= Group (e.g. community organisation) 4= Village 5 = Other 6 = Not clear |
|  | Treatment effect estimated | What treatment effect is estimated? | 1=ITT  2=ATET  3=ATE  4=LATE |
|  | Sample size (treatment) | Initial sample size treatment group | # |
|  | Sample size (control) | Initial sample size control group | # |
|  | Sample size (total) | Initial sample size total | # |
|  | Observations (treatment) | Number of treatment observations after attrition / follow up | # |
|  | Observations (control) | Number of control observations after attrition / follow up | # |
|  | Observations (total) | Total number of control observations after attrition / follow up | # |
| Outcome data - if continuous (Means and SDs) | Baseline outcome treatment | State result of baseline outcome for treatment group | # |
|  | SD Baseline outcome treatment | State SD of baseline outcome measure for treatment group | # |
|  | Baseline outcome control | State result of baseline outcome for control group | # |
|  | SD Baseline outcome control | State SD of baseline outcome measure for control group | # |
|  | Outcome in treatment post intervention | State result of post intervention outcome for treatment group | # |
|  | SD Outcome in treatment post intervention | State SD of post intervention outcome measure for treatment group | # |
|  | Outcome in control post intervention | State result of post intervention outcome for control group | # |
|  | SD Outcome in control post intervention | State SD of post intervention outcome measure for control group | # |
|  | Outcome in treatment 1st follow up | State result of 1st follow up outcome measure for treatment group | # |
|  | SD Outcome in treatment 1st follow up | State SD 1st follow up outcome measure for treatment group | # |
|  | Outcome in control 1st follow up | State result of 1st follow up outcome measure for treatment group | # |
|  | SD Outcome in control 1st follow up | State SD 1st follow up outcome measure for treatment group | # |
| Outcome data - If continuous (Mean difference and SD / SE at follow up) | Mean difference at follow up | State mean difference | # |
|  | SD at follow up | State SD at follow up | # |
|  | SE | State SE | # |
| Outcomes data - if dichotomous (Proportions r) | Baseline number with outcome in treatment | State result of baseline outcome for treatment group | # |
|  | Proportion with outcome at baseline in treatment | State proportion with outcome at baseline in treatment | # |
|  | Baseline number with outcome in control | State result of baseline outcome for treatment group | # |
|  | Proportion with outcome at baseline in control | State proportion with outcome at baseline in control | # |
|  | Number with outcome in treatment post intervention | State number with outcome post intervention for treatment group | # |
|  | Proportion with outcome in treatment group post intervention | State proportion with outcome post intervention in control group | # |
|  | Number with outcome in control post intervention | State number with outcome post intervention for control group | # |
|  | Proportion with outcome in control group post intervention | State proportion with outcome post intervention in control group | # |
|  | Number with outcome in treatment 1st follow up | State number with outcome at 1st follow up for treatment group | # |
|  | Proportion with outcome in treatment group 1st follow up | State proportion with outcome at 1st follow up in treatment group | # |
|  | Number with outcome in control 1st follow up | State number with outcome at 1st follow up for control group | # |
|  | Proportion with outcome in control group 1st follow up | State proportion with outcome at 1st follow up in control group | # |
| Regression data | OLS | OLS used? | 1=Yes 2=No |
|  | Logistic | Logistic used? | 1=Yes 2=No |
|  | Type of logistic | What type of logistic regression? | 1=binomial 2=multinomial |
|  | GLS/WLS | GLS or WLS used? | 1=Yes 2=No |
|  | other regression types | Other regression type used? Specify | open answer |
|  | continuous outcome | Is the outcome continuous? | 1=Yes 2=No |
|  | dichotomous outcome | Is the outcome dichotomous? | 1=Yes 2=No |
|  | multiple outcome categories | Does the outcome have more than 2 categories? | 1=Yes 2=No 3=Continuous |
|  | type of coefficient | What is the coefficient type? | 1=raw 2=standardized 3=other |
|  | coefficient | What is the coefficient estimate? | # |
|  | pooled standard deviation of outcome | What is the pooled standard deviation of the outcome? | # |
|  | standard error | What is the standard error of the coefficient estimate? | # |
|  | t test | What is the t statistic associated with the focal predictor? | # |

**Appendix B: Draft critical appraisal tool**

**This tool classifies study design and assesses risk of bias for quantitative impact evaluations (that is, studies included to answer questions 1 and 2).**

|  | Description | Question | Coding |
| --- | --- | --- | --- |
| ID | Unique study identification # | Study | For example, SC001 |
|  | Paper | Surname / year of first author of paper for effect size data extraction | Open answer |
| Research methods - study design and risk of bias | Design type | What type of study design is used? | 1= Randomised controlled trial (RCT) (random assignment to households/individuals) or quasi-RCT 2= Cluster-RCT (quasi-RCT) 3= Natural experiment: randomised or as-if randomised  4= Natural experiment: regression discontinuity (RD) 5 = CBA (non-randomised assignment with treatment and contemporaneous comparison group, baseline and endline data collection) – individual repeated measurement  6= CBA pseudo panel (repeated measurement for groups but different individuals) 7= Interrupted time series (with or without contemporaneous control group)  8= Panel data, but no baseline (pre-test) 9 = Comparison group with endline data only |
|  | Methods used for analysis | Which methods are used to control for selection bias and confounding? | 1= Statistical matching (PSM, CEM, covariate matching)  2= Difference in differences (DID) estimation methods 3= IV-regression (2-stage least squares or bivariate probit) 4=Heckman selection model 5= Fixed effects regression 6= Covariate adjusted estimation  7= Propensity weighted regression 8= Comparison of means 9 = Other |
|  | Design and analysis method description | Briefly describe the study design and analysis method undertaken by the authors | Open answer |
|  | Unit of analysis | Is unit of analysis in cluster allocation addressed in standard error calculation (RCT and NRS)? | 1=Yes 2=No 3=Not reported/unclear 4=Not applicable |
|  | Method used to address differences between UoA and unit of data collection | Briefly describe methods used to adjust standard errors to account for correlation of observations within clusters (e.g. cluster-robust standard errors reported) | Open answer |
|  | Type of comparison group | Indicate type of comparison group | 1=No intervention (service delivery as usual) 2=Other PITA intervention 3=Pipeline (wait-list) control (still service delivery as usual) |
|  | Assignment mechanism | 1: Mechanism of assignment: was the allocation or identification mechanism random or as good as random? | 1= Yes, 2 = Probably Yes, 3 = Probably No, 4 = No, 8 = Unclear |
|  | Assignment justification | Justification for coding decision  (Include a brief summary of justification for rating, mentioning your response to all sub questions, cite relevant pages) | Open answer |
|  | Confounding | Group equivalence: was the method of analysis executed adequately to ensure comparability of groups throughout the study and prevent confounding | 1= Yes, 2 = Probably Yes, 3 = Probably No, 4 = No, 8 = Unclear |
|  | Confounding justification | Justification for coding decision  (Include a brief summary of justification for rating, mentioning your response to all sub questions, cite relevant pages) | Open answer |
|  | Selection bias | Was any differential selection into or out of the study (attrition bias) adequately resolved? | 1= Yes, 2 = Probably Yes, 3 = Probably No, 4 = No, 8 = Unclear |
|  | Selection bias justification | Justification for coding decision  (Include a brief summary of justification for rating, mentioning your response to all sub questions, cite relevant pages) | Open answer |
|  | Spill-overs, cross-overs and contamination | 2: Spill-overs, cross-overs and contamination: was the study adequately protected against spill-overs, cross-overs and contamination? | 1= Yes, 2 = Probably Yes, 3 = Probably No, 4 = No, 8 = Unclear |
|  | Spill-overs justification | Justification for coding decision  (Include a brief summary of justification for rating, mentioning your response to all sub questions, cite relevant pages) | Open answer |
|  | Motivation bias | Was the process of being observed free from motivation bias (e.g. Hawthorne effects)? | 1= Yes, 2 = Probably Yes, 3 = Probably No, 4 = No, 8 = Unclear |
|  | Motivation justification | Justification for coding decision  (Include a brief summary of justification for rating, mentioning your response to all sub questions, cite relevant pages) | Open answer |
|  | Outcome reporting | 3: Outcome reporting: was the study free from selective outcome reporting? | 1= Yes, 2 = Probably Yes, 3 = Probably No, 4 = No, 8 = Unclear |
|  | Outcome reporting | Justification for coding decision  (Include a brief summary of justification for rating, mentioning your response to all sub questions, cite relevant pages) | Open answer |
|  | Analysis reporting | 4: Analysis reporting: was the study free from selective analysis reporting? | 1= Yes, 2 = Probably Yes, 3 = Probably No, 4 = No, 8 = Unclear |
|  | Analysis reporting | Justification for coding decision  (Include a brief summary of justification for rating, mentioning your response to all sub questions, cite relevant pages) | Open answer |
|  | Performance bias | 5: Performance bias: was the process of being observed free from motivation bias? | 1= Yes, 2 = Probably Yes, 3 = Probably No, 4 = No, 8 = Unclear |
|  | Performance bias | Justification for coding decision  (Include a brief summary of justification for rating, mentioning your response to all sub questions, cite relevant pages) | Open answer |
|  | Other bias | 6: Other risks of bias: Is the study free from other sources of bias? Including around measurement of the intervention | 1= Yes, 2 = Probably Yes, 3 = Probably No, 4 = No, 8 = Unclear |
|  | Other bias | Justification for coding decision  (Include a brief summary of justification for rating, mentioning your response to all sub questions, cite relevant pages) | Open answer |
|  | Blinded participants | Blinding of participants? | 1=Yes 2=No 9= N/A |
|  | Blinded observers | Blinding of outcome assessors? | 1=Yes 2=No 9= N/A |
|  | Blinded analysts | Blinding of data analysts? | 1=Yes 2=No 9= N/A |
|  | Method used to blind | Describe method(s) used to blind | Open answer (including describe method of placebo control) |

**Appendix C: Search strategy**

1. **CAB Abstracts (Ebsco) – Searched 20^th^ November 2020**

S13 S11 AND S12 Limiters - Publication Year: 2000-2020

**1,715**

S12 TI ( ( "quasi experiment*" OR quasi-experiment* OR "random* control* trial*" OR "random* trial*" OR rct* OR ( random* N3 allocat* ) OR evaluat* OR impact* OR assess* OR dif-dif OR "double difference" OR difference-in-difference OR "difference in difference" OR "statistical matching*" OR "propensity score matching" OR "covariate matching" OR "coarsened-exact matching" OR "propensity-weighted" OR "multiple regression" OR "statistical regression" OR "regression discontinuity*" OR "cohort analysis" OR "quantitative method*" OR "program* evaluation" OR "interrupted time series" OR ( before N5 after ) OR ( pre N5 post ) OR ( ( pretest OR "pre test" ) AND ( posttest OR "post test" ) ) OR ( "fixed effect*" N3 ( model OR estimation ) ) OR "instrumental variable*" OR "synthetic control" OR ( ( quantitative OR "comparison group*" OR counterfactual OR "counter factual" OR counter-factual OR experiment* ) N3 ( design OR study OR analysis ) ) ) ) OR AB ( ( "quasi experiment*" OR quasi-experiment* OR "random* control* trial*" OR "random* trial*" OR rct* OR ( random* N3 allocat* ) OR evaluat* OR impact* OR assess* OR dif-dif OR "double difference" OR difference-in-difference OR "difference in difference" OR "statistical matching*" OR "propensity score matching" OR "covariate matching" OR "coarsened-exact matching" OR "propensity-weighted" OR "multiple regression" OR "statistical regression" OR "regression discontinuity*" OR "cohort analysis" OR "quantitative method*" OR "program* evaluation" OR "interrupted time series" OR ( before N5 after ) OR ( pre N5 post ) OR ( ( pretest OR "pre test" ) AND ( posttest OR "post test" ) ) OR ( "fixed effect*" N3 ( model OR estimation ) ) OR "instrumental variable*" OR "synthetic control" OR ( ( quantitative OR "comparison group*" OR counterfactual OR "counter factual" OR counter-factual OR experiment* ) N3 ( design OR study OR analysis ) ) ) ) OR SU ( ( "quasi experiment*" OR quasi-experiment* OR "random* control* trial*" OR "random* trial*" OR rct* OR ( random* N3 allocat* ) OR evaluat* OR impact* OR assess* OR dif-dif OR "double difference" OR difference-in-difference OR "difference in difference" OR "statistical matching*" OR "propensity score matching" OR "covariate matching" OR "coarsened-exact matching" OR "propensity-weighted" OR "multiple regression" OR "statistical regression" OR "regression discontinuity*" OR "cohort analysis" OR "quantitative method*" OR "program* evaluation" OR "interrupted time series" OR ( before N5 after ) OR ( pre N5 post ) OR ( ( pretest OR "pre test" ) AND ( posttest OR "post test" ) ) OR ( "fixed effect*" N3 ( model OR estimation ) ) OR "instrumental variable*" OR "synthetic control" OR ( ( quantitative OR "comparison group*" OR counterfactual OR "counter factual" OR counter-factual OR experiment* ) N3 ( design OR study OR analysis ) ) ) )

2,871,753

S11 S1 OR S2 OR S3 OR S6 OR S7 OR S8 OR S9 OR S10

4,043

S10 TI ( ("energy audit*" or (behavio* N2 chang*) or (information N2 (provid* or provision))) N3 "energy efficien*" N4 (building* or "built environment*" or home or homes or housing or house or houses or household* or residen* or domestic or dwelling* or domicil* or occupan*cor apartment* or condominium*)) OR AB ( ("energy audit*" or (behavio* N2 chang*) or (information N2 (provid* or provision))) N3 "energy efficien*" N4 (building* or "built environment*" or home or homes or housing or house or houses or household* or residen* or domestic or dwelling* or domicil* or occupan* or apartment* or condominium*)) OR SU ( ("energy audit*" or (behavio* N2 chang*) or (information N2 (provid* or provision))) N3 "energy efficien*" N4 (building* or "built environment*" or home or homes or housing or house or houses or household* or residen* or domestic or dwelling* or domicil* or occupan* or apartment* or condominium*))

1

S9 TI ( (("fossil fuel*" N3 independen*) or "zero energy" or "green building" or ("whole building*" N3 simulat*) or ("energy productivity" N3 indicator*) or technolog* or (retrofit N2 (level* or program*)) or "air condition*" or HVAC* or "heating ventilation" or lighting or lightbulb* or "light bulb*" or (LED N2 light*)) N4 "energy efficien*" ) OR AB ( (("fossil fuel*" N3 independen*) or "zero energy" or "green building" or ("whole building*" N3 simulat*) or ("energy productivity" N3 indicator*) or technolog* or (retrofit N2 (level* or program*)) or "air condition*" or HVAC* or "heating ventilation" or lighting or lightbulb* or "light bulb*" or (LED N2 light*)) N4 "energy efficien*" ) OR SU ( (("fossil fuel*" N3 independen*) or "zero energy" or "green building" or ("whole building*" N3 simulat*) or ("energy productivity" N3 indicator*) or technolog* or (retrofit N2 (level* or program*)) or "air condition*" or HVAC* or "heating ventilation" or lighting or lightbulb* or "light bulb*" or (LED N2 light*)) N4 "energy efficien*" )

73

S8 TI ( ((furnace* or boiler* or "energy burden" or infiltration or (electric* N3 "peak demand*")) N3 (replac* or reduc*)) ) OR AB ( ((furnace* or boiler* or "energy burden" or infiltration or (electric* N3 "peak demand*")) N3 (replac* or reduc*)) ) OR SU ( ((furnace* or boiler* or "energy burden" or infiltration or (electric* N3 "peak demand*")) N3 (replac* or reduc*)) )

2,289

S7 TI ( ((weatheri* or unweatheri* or replac* or insulat* or upgrad* or "high performance") N3 (window* or door* or attic* or loft* or wall or walls or floor* or appliance* or "heat pump*" )) ) OR AB ( ((weatheri* or unweatheri* or replac* or insulat* or upgrad* or "high performance") N3 (window* or door* or attic* or loft* or wall or walls or floor* or appliance* or "heat pump*")) ) OR SU ( ((weatheri* or unweatheri* or replac* or insulat* or upgrad* or "high performance") N3 (window* or door* or attic* or loft* or wall or walls or floor* or appliance* or "heat pump*")) )

386

S6 S4 AND S5

42

S5 DE "housing" OR DE "cooperative housing" OR DE "dwellings" OR DE "homes" OR DE "public housing" OR DE "rural housing" OR DE "single family housing" OR DE "households" OR DE "housing costs" OR DE "living standards"

45,255

S4 ( DE "electrical energy" OR DE "renewable energy" OR DE "thermal energy" OR DE "energy conservation" OR DE "energy consumption" OR DE "energy sources" ) AND ( DE "efficiency" OR DE "combustion efficiency" OR DE "use efficiency" OR DE "thermal efficiency" )

3,503

S3 TI ( ("energy efficien*" N3 (retrofit* or improv* or measur* or program* or intervention* or audit*)) ) OR AB ( ("energy efficien*" N3 (retrofit* or improv* or measur* or program* or intervention* or audit*)) ) OR SU ( ("energy efficien*" N3 (retrofit* or improv* or measur* or program* or intervention* or audit*)) )

1,273

S2 TI ( ((weatheriz* or weatheris*) N3 (building* or "built environment*" or home or homes or housing or house or houses or household* or residen* or domestic or dwelling* or domicil* or occupan* or apartment* or condominium*)) ) OR AB ( ((weatheriz* or weatheris*) N3 (building* or "built environment*" or home or homes or housing or house or houses or household* or residen* or domestic or dwelling* or domicil* or occupan* or apartment* or condominium*)) ) OR SU ( ((weatheriz* or weatheris*) N3 (building* or "built environment*" or home or homes or housing or house or houses or household* or residen* or domestic or dwelling* or domicil* or occupan* or apartment* or condominium*)) )

9

S1 TI (residential N3 "energy efficien*" N3 intervention*) OR AB (residential N3 "energy efficien*" N3 intervention*) OR SU (residential N3 "energy efficien*" N3 intervention*)

3
